# Validity, reliability, acceptability, and utility of the Social Inclusion Questionnaire User Experience (SInQUE): a clinical tool to facilitate social inclusion amongst people with severe mental health problems

**DOI:** 10.1007/s00127-019-01826-3

**Published:** 2020-02-03

**Authors:** Gillian Mezey, Sarah White, Isobel Harrison, Jennifer Bousfield, Brynmor Lloyd-Evans, Sarah Payne, Helen Killaspy

**Affiliations:** SGUL, London, UK

**Keywords:** Social inclusion, Psychometrics, mental illness, Psychosis, common mental disorder, Personality disorder

## Abstract

**Background:**

Individuals with severe mental health problems are at risk of social exclusion, which may complicate their recovery. Mental health and social care staff have, until now, had no valid or reliable way of assessing their clients’ social inclusion. The Social Inclusion Questionnaire User Experience (SInQUE) was developed to address this. It assesses five domains: social integration; productivity; consumption; access to services; and political engagement, in the year prior to first psychiatric admission (T1) and the year prior to interview (T2) from which a total score at each time point can be calculated.

**Aims:**

To establish the validity, reliability, and acceptability of the SInQUE in individuals with a broad range of psychiatric diagnoses receiving care from community mental health services and its utility for mental health staff.

**Method:**

Participants were 192 mental health service users with psychosis, personality disorder, or common mental disorder (e.g., depression, anxiety) who completed the SInQUE alongside other validated outcome measures. Test–retest reliability was assessed in a sub-sample of 30 participants and inter-rater reliability was assessed in 11 participants. SInQUE ratings of 28 participants were compared with those of a sibling with no experience of mental illness to account for shared socio-cultural factors. Acceptability and utility of the tool were assessed using completion rates and focus groups with staff.

**Results:**

The SInQUE demonstrated acceptable convergent validity. The total score and the Social Integration domain score were strongly correlated with quality of life, both in the full sample and in the three diagnostic groups. Discriminant validity and test–retest reliability were established across all domains, although the test–retest reliability on scores for the Service Access and Political Engagement domains prior to first admission to hospital (T1) was lower than other domains. Inter-rater reliability was excellent for all domains at T1 and T2.

**Conclusions:**

The component of the SInQUE that assesses current social inclusion has good psychometric properties and can be recommended for use by mental health staff.

## Background

Social exclusion occurs when an individual does not participate in key activities in their society, that they would like to participate in, for reasons that are beyond their control [1–3]. Social exclusion is multi-dimensional, multi-layered, and dynamic, and it is associated, although not synonymous, with poverty [4, 5].

Individuals with mental health problems are particularly likely to experience social exclusion [2, 4, 6–10]. Increasing the social inclusion of individuals with mental health problems may help to improve their mental health and wellbeing [2, 6, 7, 11].

Despite the fact that social inclusion is often cited as an important treatment outcome by mental health service users, it is often neglected by mental health professionals when considering care planning [7, 12]. One factor contributing to this may be the lack of a validated, robust, and acceptable measure of social inclusion for use in mental health services [2, 4, 13, 14].

There are a number of measures of social inclusion, of variable quality and suitability, such as the Social and Community Opportunities Profile (SCOPE; Huxley et al., [15]), and the Social Inclusion Scale (SIS; Secker et al., [16]). Questions about the applicability, relevance, and suitability of existing measures as indicators of social inclusion have been raised [13]. A number of measures have been developed that assess related concepts, such as social integration, social networks, and quality of life, or which represent composite measures, created from a selection of questions and subscales [13]. However, reviews of existing measures of social inclusion for individuals with mental health problems have concluded that, despite recent developments, further progress is required [14].

The Social Inclusion Questionnaire User Experience (SInQUE) is a measure of social inclusion, for use with mental health service users, which has been assessed as comprehensive and potentially suitable for cross-cultural use [13]. One advantage it has over other measures is that it enquires about both objective and subjective aspects of social inclusion, i.e., the person’s actual participation in activities (objective) as well as whether they wish to participate in those activities (subjective). The tool is thus able to inform individualised care planning by identifying areas for potential improvement.

The SInQUE was specifically developed to assist mental health professionals to assess social inclusion with their clients in the community [17]. The content of the measure was based on the UK Poverty and Social Exclusion Survey [18] which assessed the extent, consequences, and factors associated with poverty, deprivation, and social exclusion in Britain. Five relevant domains were identified: productivity, consumption, access to services, social integration, and political engagement. The SInQUE assesses an individual’s actual and desired current social inclusion, in relation to the various items that contribute to the five domains, both currently and in the year prior to their first psychiatric admission. In an earlier pilot study using the SInQUE with individuals with a diagnosis of schizophrenia or schizoaffective disorder, we found that social inclusion declined over the time since the start of their illness. Longer duration of illness and older age at first admission were associated with greater decline. Higher levels of social inclusion were associated with better quality of life [19]. The study also demonstrated good convergent and discriminant validity of the SInQUE in individuals with a diagnosis of schizophrenia or schizoaffective disorder [17].

This paper reports on the results of further psychometric testing of the SInQUE in mental health service users, with a broader range of psychiatric diagnoses, under the care of NHS community mental health services. The aims were to assess the tools: concurrent, convergent, and discriminant validity; test–retest and inter-rater reliability; acceptability to service users; and perceived utility to mental health staff. In a sub-sample of participants, we also assessed SInQUE ratings of a sibling who had not experienced any mental illness to account for the contribution of mental disorder on social inclusion, as opposed to other shared family, or social cultural factors.

## Methods

### Procedures

The study was approved by the London–Bromley Research and Ethics Committee (ref IS/LO/1778).

This was a mixed-method cross-sectional study, including the collection of retrospective data. Quantitative data were collected through individual structured interviews with service users and the siblings of a sub-sample. The case records of service users were reviewed to obtain or confirm some socio-demographic data and psychiatric contacts.

An information sheet about the study was sent to the managers and consultant psychiatrists of the community mental health services in the two trusts. Team members were asked to identify service users who met the inclusion criteria and could be approached to take part in the study. If the service user agreed to be contacted about the study, their name was passed to one of the researchers (JB, IH), who then arranged to meet them, usually at the community team base or at their home. The service user was given a participant information sheet about the study and had an opportunity to ask questions about the study before providing written informed consent.

All service user participants were asked to nominate, where possible, a sibling with no history of mental health problems who could be contacted by the researchers. Nominated siblings were sent an information sheet about the study and a consent form to return to the research team in a pre-paid envelope. They were given an opportunity to discuss the study and ask questions in a follow-up telephone call with the researcher. Siblings were offered a choice of being interviewed face-to-face or by telephone.

During April and May 2017, mental health professionals from the participating teams were invited, by e-mail, to contribute to a focus group. The purpose of the focus groups was explained and written consent obtained from participating staff. Four focus groups (two in each participating Trust) each consisting of four mental health professionals were held conducted to explore staff views of the SInQUE, including its potential utility. The groups were recorded and transcribed, and the transcripts were anonymised. Each focus group took approximately 1 h and was facilitated by both researchers (JB, IH). The data were subjected to qualitative analysis.

Rates of recruitment and completion of the SInQUE were assessed as an indicator of its acceptability to service users.

All service user and sibling participants received £20 for each research interview in recognition of their time.

### Participants and setting

Recruitment took place between December 2015 and May 2017.

*Service users* Service user participants were recruited from community mental health services in South West London and St Georges NHS Mental Health Trust (SWLSTG) and Camden and Islington NHS Foundation Trust (C & I). Participating teams included community mental health teams, community rehabilitation teams, complex depression and trauma teams, personality disorder services, assertive outreach teams, early intervention for psychosis services, and community forensic services.

All participants had to be over 18 years old and be able to speak and understand English. Additional inclusion criteria were: a primary diagnosis of a psychotic illness (e.g., schizophrenia, schizoaffective disorder, or bipolar affective disorder), common mental disorder (depression, anxiety, obsessive compulsive disorder, and posttraumatic stress disorder) or personality disorder; currently receiving treatment from one of the community teams listed above; at least one previous inpatient admission or a period of care from a Crisis Resolution/Home Treatment Team and a period of at least 3 months living in the community, since last inpatient admission.

*Staff* Staff participating in the focus groups were purposively sampled to include a range of mental health professional disciplines.

### Measures

Study data were all collected through a face-to-face structured interview with a researcher, using validated self-report scales, to assess the psychometric properties.

#### Service users

*Socio-demographic data and psychiatric history* Participants were assessed through face-to-face interview to obtain age; gender; ethnicity; current civil status; educational attainment; current employment status; diagnosis (ICD-10); duration of contact with psychiatric services. Case notes were reviewed to gain details of contacts with staff in the last year.

*SInQUE* [17] The SInQUE is completed through a structured interview and comprises items that provide scores on five domains: productivity (PRO), consumption (CON), access to services (SA), social integration (SI), and political engagement (POL). Ratings are recorded in relation to the year prior to the service user’s first admission to hospital, or treatment from a Home Treatment/crisis team (T1-32 items) and in the past year (T2-58 items). First admission or home treatment for a mental health crisis is used as a proxy for the person’s first contact with mental health services as it is likely to have been a memorable event. Some items which are in T2 are not included in T1, to reduce the possibility of recall bias regarding events and experiences which may have happened many years ago. For example, T1 does not include a question about the number of people outside their care team in whom they were able to confide, or how many neighbours they knew by name, or whether they would have liked to have had more friends. These questions are, however, all included in T2.

Total T1 and T2 scores as well as individual domain scores are generated, with higher scores denoting greater social inclusion. The SInQUE takes approximately 30 min to complete.

*Social Outcomes Index (SIX)* [20] The SIX comprises four questions about social outcomes. It provides a total maximum score of 6 with higher scores denoting better outcomes: employment (0–2); accommodation (0–2); partnership/family support (0–1); and friendship (0–1). SIX scores were assessed currently (T2). Ratings were used to assess the SInQUE’s concurrent validity. It takes less than 5 min to administer.

*Manchester short assessment of quality of life (MANSA)* [21] The MANSA consists of 17 items that assess overall quality of life and factors contributing to this (life domains). Items are rated from 1 (could not be worse) to 7 (could not be better), and a total mean score (from 1 to 7) is generated. The MANSA takes around 10 min to complete and was rated in relation to T2 only. Ratings were used in our assessment of the SInQUE’s convergent and discriminant validity.

*Camberwell assessment of need short appraisal schedule (CANSAS)* [22] The CANSAS is a structured interview assessing 22 areas of need. Responses are rated on a three-point scale (0 = no serious need; 1 = no need or moderate need due to continuing intervention [met need]; 2 = serious need whether receiving intervention or not [unmet need]). An overall total score of met and unmet needs is generated. The CANSAS takes approximately 10 min to complete and was assessed in relation to T2 only. Ratings were used in our assessment of the SInQUE’s convergent and discriminant validity.

*Brief psychiatric rating scale (BPRS)* [23] The BPRS is a researcher rated symptom rating tool. Each of the 18 items are rated on a scale of 1 = not present through to 7 = severe and a total mean score from 1 to 7 is generated. The BPRS was rated in relation to T2 only and takes approximately 10 min to complete. Ratings were used in our assessment of the SInQUE’s convergent and discriminant validity.

*Discrimination and stigma scale (DISC -12)* [24] This is a self-report scale assessing the person’s experience of stigma and discrimination comprising two subscales: unfair treatment (21 items) and stopping self (4 items). Items are rated on a 4 point Likert scale: not at all (0); a little (1); moderately (2); a lot (3). For the purpose of this study, we assessed current (past year) service user participant experiences. Ratings were used in our assessment of the SInQUE’s discriminant validity. It takes about 10 min to complete.

#### Siblings

*Socio-demographic data* were recorded through face-to-face or telephone interview including age, gender, ethnic group, marital status, educational achievement, and current employment. The *SInQUE* was also completed and ratings used in our assessment of its concurrent validity.

#### Staff

The focus groups were facilitated by the researchers, using a topic guide which was designed and developed using input from our service user research advisory panels. The focus groups explored the concept of social exclusion in general, current measures, and practices for assessing and addressing social exclusion amongst people with mental health problems, as well as staff perceptions of the comprehensiveness, utility, and applicability of the SInQUE. Staff participants were also asked whether they would use the tool in their practice, and if so, in which contexts or settings, and with which service user groups.

### Quantitative data analysis

#### Sample size and power

We aimed to recruit 200 participants (80 with psychosis, 80 with common mental disorder, and 40 with personality disorder), an adequate sample size to estimate the SInQUE’s validity and reliability using correlation coefficients ≥  ± 0.3 with confidence limits ≤  ± 0.125. This sample size also provided 80% power to detect between group-effect sizes of ≥ 0.55 at a 5% significance level.

#### Statistical analysis

*Concurrent validity* of the SInQUE was investigated in two ways: first by correlating participants’ T2 SInQUE total and domain scores with T2 SIX scores; second, by correlating participants’ T1 and T2 SInQUE total and domain scores, and comparing any change in SInQUE total and domain scores from T1 to T2, with their non-affected siblings’ scores. Paired sample *t* tests were used to assess change in the social integration (SI), consumption (CON), and access to services (SA) domain scores. Wilcoxon signed-rank tests were used to assess change in productivity (PRO) and political engagement (POL) domain scores as these domains had narrow ranges and skewed distributions. We hypothesised that there would be no statistically significant differences in the T1 SInQUE scores of service users and their siblings, but service users would have lower T2 SInQUE scores compared with their siblings.

*Convergent validity* was investigated by assessing the correlation of the T2 SInQUE total and domain scores with current quality of life (MANSA), unmet needs (CANSAS), and symptoms (BPRS). We hypothesised that SInQUE total and domain scores would be positively correlated with quality of life (MANSA) and negatively correlated with unmet needs (CANSAS) and symptoms (BPRS).

*Discriminant validity* was assessed using the correlation of participants’ T1 SInQUE total and domain scores with current quality of life (MANSA), symptoms (BPRS), and stigma/discrimination (DISC). We hypothesised that social inclusion prior to first psychiatric admission would not be correlated with current quality of life, symptoms, and experience of stigma and discrimination.

All validity analyses were carried out in relation to the whole sample and by diagnostic group, with the exception of those involving sibling ratings where small numbers precluded this.

The statistical significance of the correlation coefficients (*r*) is not informative when the sample size is large so *r* ≥ 0.3 was considered clinically significant in this validity analysis. This threshold was chosen as it is considered a medium-effect size [25]. In the discriminant validity analysis by diagnostic group, the sample sizes for the common mental disorder and personality disorder groups were 33 and 32, respectively. In interpreting these *r*’s, those above 0.35 were considered clinically significant, the threshold increased due to the smaller sample size. Pearson correlation coefficients are used throughout the validity analysis.

*Test–retest reliability* of the SInQUE was assessed with 30 participants (10 from each diagnostic group) who completed the SInQUE on a second occasion with the researcher, between 5 and 10 days after the first interview.

*Inter-rater reliability* of the SInQUE was assessed by both researchers, by rating 11 participant interviews (one of whom led the interview and the other completed a second rating).

Test retest and inter-rater reliability were estimated by intra-class correlation coefficients (ICCs) and 95% CI for the SInQUE total and domain scores at T1 and T2, with an ICC of ≥ 0.7 considered acceptable.

Acceptability of the SInQUE was assessed through rates of participation and completion of the measure by service users and through staff feedback at the focus groups.

### Qualitative data analysis

Focus groups were coded by both researchers. Thematic analysis was conducted using qualitative analysis software (Nvivo 11). Thematic analysis is a widely used method to identify patterns in the data [26]. In exploring the acceptability and perceived usefulness of the SInQUE, we used a deductive analysis, whereby our initial data were organised according to the main topics covered in the topic guide.

All transcripts were double coded by the two research assistants and any inconsistencies resolved by consultation with the wider research team.

## Results

### Response

The researchers contacted all 39 eligible community mental health teams across both Trusts. Twenty two of the teams referred service users to the study (9 C&I; 13 SWLSTG). A total of 238 service users were referred from these 22 teams. Of these, 6 did not meet the inclusion criteria, 11 could not be contacted, 28 declined to take part, and 1 withdrew post-consent. The response rate was, therefore, 192/238 (80.7%).

### Service user participant characteristics

Service user socio-demographic and clinical characteristics are shown in Table 1.

**Table 1:** Socio-demographic characteristics and clinical history of sample by diagnostic group; values are *n* (%) unless otherwise stated

	Diagnostic group							
	Psychosis		Common mental disorder		Personality disorder		Total	
	106		49		37		192	
Socio-demographic characteristics								
Age, mean (SD), Min–max	43.7 (10.9) 23–74		45.1 (11.6) 18–71		35.1 (10.0) 19–52		42.4 (11.4) 18–74	
Gender								
Male	60	56.6%	16	32.7%	9	24.3%	85	44.3%
Female	46	43.4%	33	67.3%	28	75.7%	107	55.7%
Ethnicity								
White	60	56.6%	39	79.5%	30	81.1%	129	67.2%
Black	31	29.3%	5	10.0%	1	2.7%	37	19.3%
Mixed race	12	11.4%	1	2.0%	3	8.1%	16	8.3%
Asian	2	1.9%	4	8.0%	0	0.0%	6	3.1%
Other	1	0.9%	0	0.0%	3	8.1%	4	2.1%
Marital status								
Single, never married/cohabited	82	77.4%	31	63.3%	33	89.2%	146	76.0%
Single, divorced/separated	13	12.3%	11	22.4%	3	8.1%	27	14.1%
Single, widow/er	2	1.9%	0	0.0%	0	0.0%	2	1.0%
Currently married/partnered/civil partnered	8	7.5%	7	14.3%	1	2.7%	16	8.3%
Highest level of education reached								
No qualification	33	31.1%	7	14.3%	6	16.2%	46	24.0%
General certificate special education [GCSE (or equivalent)]	27	25.5%	15	30.6%	16	43.2%	58	30.2%
A level	24	22.6%	13	26.5%	9	24.3%	46	24.0%
University degree (or equivalent)	21	19.8%	13	26.5%	6	16.2%	40	20.8%
Employment status								
Paid employment	9	8.5%	6	12.2%	8	21.6%	23	12.0%
Sheltered employment	2	1.9%	0	0.0%	0	0.0%	2	1.0%
Training/education	2	1.9%	1	2.0%	2	5.4%	5	2.6%
Unemployed	88	83.0%	40	81.6%	27	73.0%	155	80.7%
Retired	4	3.8%	2	4.1%	0	0.0%	6	3.1%
Other	1	1.0%	0	0.0%	0	0.0%	1	0.5%
Has children								
Yes—lives with	3	2.8%	8	16.3%	2	5.4%	13	6.8%
Yes—lives apart	26	24.5%	14	28.6%	10	27.0%	50	26.0%
No	77	72.6%	27	55.1%	24	66.7%	128	67.2%
Living alone								
Yes	49	46.2%	22	44.9%	17	45.9%	88	45.8%
No	57	53.8%	27	55.1%	20	54.1%	104	54.2%
If no, who with?								
Friends	1	1.8%	3	11.1%	1	5.0%	5	4.8%
Family members	22	38.6%	23	85.2%	14	70.0%	59	56.7%
Mental health service users	34	59.6%	1	3.7%	3	15.0%	38	36.5%
Other	0	0%	0	0%	2	10.0%	2	1.9%
Clinical history								
Mean length of contact with mental health services (years)	19.4 (10.7) 1–53		15.5 (11.2) 1–45		14.4 (8.3) 2–38		17.5 (10.6) 1–53	
Mean time since last hospital admission (months)	66.5 (70.2) 4–312		76.2 (102.2) 5–360		51.9 (71.6) 3–288		65.2 (76.6) 3–360	
Index event, *n* (%)								
Inpatient admission	106 (100%)		23 (46.9%)		30 (81.1%)		159 (82.8%)	
Crisis team treatment	0 (0%)		10 (20.4%)		2 (5.4%)		12 (6.3%)	
None	0 (0%)		16 (32.7%)		5 (13.5%)		21 (10.9%)	
Mean (SD), range previous admissions	5.6 (5.0) 1–30		1.2 (2.1) 0–12		7.2 (12.0) 0–50		4.8 (6.9) 0–50	
Mean (SD), range previous involuntary admissions	3.7 (4.4) 0–25		0.4 (0.8) 0–4		3.5 (7.3) 0–29		2.8 (4.8) 0–29	
Mean (SD, range) face-to-face contacts with care co-ordinator/psychiatrist in last year	22.6 (17.2) 4–104		27.2 (16.9) 3–52		31.5 (24.3) 4–104		25.6 (19.0) 3–104	

One hundred and ninety-two service users (81% of those referred) were recruited; 106 (55%) into the psychosis group, 49 (26%) into the common mental disorders group, and 37 (19%) into the personality disorder group. Service users’ mean age was 42.2 years [standard deviation (SD) 11.4]. The majority were white (67%), single (76%), and unemployed (80.7%). Over half (107; 56%) were female; with women predominating in the personality disorder and common mental disorder groups. Service users with psychosis were more likely to be from a Black or Ethnic Minority than the other two diagnostic groups (43.4% vs 20.5% common mental disorder, 18.9% personality disorder), more likely to have no educational qualifications (31.1% vs 14.3% and 16.2%) and more commonly lived with other service users (59.6 vs 3.7% and 15.0%) than family compared to the other two groups (38.6% vs 85.2% and 70%).

Service users had been in contact with mental health services for 17.5 years on average (SD 10.6), with a mean of 4.8 admissions (SD 6.9). Those with psychosis had slightly longer duration of contact (mean 19.4 years, SD 10.7) and more previous involuntary admissions than the other two diagnostic groups. Those with personality disorder had more previous admissions than the other two groups, and more face-to-face contacts with their care co-ordinator or psychiatrist over the previous year.

### Sibling characteristics

The mean age of the 28 sibling participants was 40.3 years (SD 12.89). 16 (57%) were female and 22 (79%) white. 14 (50%) were single or never married. 1 in 3 of them (75%) had achieved at least ‘A’ level qualifications, and 21 (75%) were in paid employment, training, and education currently.

### Staff characteristics

Sixteen mental health professionals (4 male; 12 female) took part in the four focus groups. Staff included social workers, nurses, occupational therapists, psychologists, psychiatrists, and a team manager.

### Measures of validity

The results of the concurrent validity analyses are shown in Tables 2 and 3. Table 2 shows that the SIX was correlated with the T2 SInQUE total score, as well as with the social integration and productivity domain scores in the full sample and each of the three diagnostic groups. In the whole sample and the psychosis group, SIX was correlated with the T2 SInQUE consumption domain score, and, in the common mental disorder group, there was correlation with the T2 SInQUE political domain score.

**Table 2:** Concurrent validity—correlation between SINQUE domains and SIX at T2

	Psychosis (*n* = 106)	CMD (*n* = 49)	PD (*n* = 37)	Total (*n* = 192)
Social integration	0.47	0.50	0.68	0.50
Consumption	0.50	0.17	0.24	0.37
Productivity	0.45	0.38	0.51	0.45
Access to services	0.17	-0.12	0.12	0.13
Political engagement	0.13	0.38	0.03	0.16
Total SInQUE	0.65	0.41	0.62	0.57

*r* > 0.3 considered significant (in green)

**Table 3:** Concurrent validity—analysis of service users with their paired siblings

	*n*	Service user mean (SD)	Sibling mean (SD)	Difference (95% CI)	Significance^b^
T1					
Social integration	24	20.0 (6.7)	23.1 (6.8)	− 3.0 (− 5.9, − 0.2)	**0.038**
Consumption	24	17.4 (4.2)	15.8 (4.3)	1.6 (0.1, 3.1)	**0.042**
Productivity^a^	23	4.0 (3.3 to 4.0)	4.0 (3.0 to 4.0)	0.0 (0.0, 0.0)	0.734
Access to services	24	5.2 (1.2)	4.9 (1.5)	0.3 (− 0.3, 0.9)	0.285
Political engagement^a^	24	1.0 (0.0 to 1.0)	1.0 (0.0 to 1.5)	0.0 (0.0, 1.0)	0.484
Total SInQUE	24	46.3 (10.5)	47.6 (10.7)	− 1.3 (− 5.6, 2.9)	0.522
T2					
Social integration	28	22.9 (6.2)	27.8 (5.4)	− 4.8 (− 7.1, − 2.5)	** < 0.001**
Consumption	28	17.6 (3.7)	18.8 (3.9)	− 1.3 (− 2.6, 0.1)	0.074
Productivity^a^	28	0.0 (0.0 to 2.8)	4.0 (1.8 to 4.0)	− 4.0 (− 4.0, − 1.0)	** < 0.001**
Access to services	28	3.0 (1.1)	3.2 (1.1)	− 0.2 (− 0.7, 0.4)	0.518
Political engagement^a^	28	1.0 (1.0 to 1.0)	1.0 (1.0 to 1.0)	0.0 (0.0, 0.0)	1.0
Total SInQUE	28	45.5 (10.6)	53.9 (9.9)	− 8.3 (− 12.2, − 4.5)	** < 0.001**
Change from T1 to T2 (T1–T2)					
Social integration	24	− 3.0 (6.8)	− 3.9 (6.4)	0.9 (− 4.9, 3.2)	0.658
Consumption	24	− 0.4 (4.1)	− 2.6 (4.5)	2.2 (0.4, 4.0)	**0.019**
Productivity^a^	23	3.0 (0.3 to 4.0)	0.0 (− 1.0 to 0.0)	2.0 (0.0, 4.0)	** < 0.001**
Access to services	24	2.1 (1.4)	1.7 (1.3)	0.4 (− 0.3, 1.1)	0.248
Political engagement^a^	24	0.0 (− 1.0 to 0.0)	0.0 (− 1.0 to 0.5)	0.0 (0.0, 0.0)	0.628
Total SInQUE	24	0.1 (11.7)	− 4.8 (8.4)	4.9 (0.6, 9.3)	**0.027**

^a^Values are median (LQ–UQ), median difference (95% CI around median difference), Wilcoxon signed-rank test (exact *p* value)

^b^Paired sample *t* test unless otherwise stated

The bold values refer to *p* < 0.05

The comparison of service users’ SInQUE scores with their siblings’ scores is reported in Table 3. At T1, there was no significant difference between service users and siblings on the total SInQUE score and the productivity, service access, and political engagement domains. However, service users scored significantly lower than their siblings on the social integration and consumption domains. At T2, siblings scored significantly higher on the social integration and productivity domains as well as the total SINQUE. There was a statistically significant difference between service users and their siblings in the change in scores from T1 to T2 for the consumption and productivity domains; service users’ productivity scores reduced, whereas their siblings’ remained stable. Service users’ consumption scores remained stable between T1 and T2, whereas their siblings’ increased. With respect to the total SInQUE score, the service users remained stable as opposed to their siblings whose total SInQUE increased by nearly five points, the difference being statistically significant.

The results of the convergent validity analyses are shown in Table 4. Across the whole service user sample, the T2 SInQUE total score and social integration domain score were strongly correlated with service user ratings of quality of life (MANSA), but other SInQUE domains were not. There was also a correlation between quality-of-life scores and the T2 SInQUE social integration domain score in all three diagnostic groups. However, the consumption domain scores were only correlated with MANSA scores in the common mental disorders and personality disorder groups. Across the whole sample, there were only marginal correlations between T2 SInQUE total and domain scores and unmet needs (CANSAS) and symptoms (BPRS).

**Table 4:** Convergent validity—correlation between T2 SINQUE domains and measures of quality of life (MANSA), psychiatric symptoms (BPRS), and unmet needs (CANSAS)

	Quality of life	Psychiatric symptoms	Unmet needs
Total sample (*n* = 192)			
Social integration	**0.59**	− 0.28	− 0.25
Consumption	0.28	− 0.25	− 0.14
Productivity	0.09	− 0.05	− 0.14
Access to services	0.00	− 0.02	− 0.02
Political engagement	− 0.04	− 0.18	− 0.02
Total SInQUE	0.50	− 0.29	− 0.25
Psychosis sample (*n* = 106)			
Social integration	**0.68**	− **0.31**	− 0.22
Consumption	0.29	− 0.18	− 0.07
Productivity	0.17	− 0.07	− 0.17
Access to services	0.18	− 0.07	− 0.16
Political engagement	− 0.06	− 0.07	0.09
Total SInQUE	0.57	− 0.27	− 0.21
CMD sample (*n* = 49)			
Social integration	**0.59**	− **0.34**	− **0.43**
Consumption	**0.31**	− **0.52**	− 0.24
Productivity	0.22	0.15	− 0.28
Access to services	− 0.04	− 0.22	− 0.08
Political engagement	0.01	− 0.25	0.00
Total SInQUE	**0.53**	− **0.43**	− **0.37**
PD sample (*n* = 37)			
Social integration	**0.56**	− 0.11	− 0.12
Consumption	**0.41**	− 0.25	− 0.20
Productivity	0.20	− **0.37**	− 0.21
Access to services	− 0.05	0.06	0.17
Political engagement	− 0.07	− **0.50**	− **0.33**
Total SInQUE	**0.56**	− 0.24	− 0.25

*r* > 0.3 considered significant (in bold)

The discriminant validity assessment is shown in Table 5. Across the whole sample, there were no significant correlations between T1 SInQUE total and domain scores and the other measures rating quality of life (MANSA), unmet needs (CANSAS), and stigma (DISC-12) currently. Four of the thirty-six correlations in the common mental disorder group and seven in the personality disorder group exceeded the 0.35 threshold.

**Table 5:** Discriminant validity—correlation between T1 SINQUE domains and measures of quality of life, psychiatric symptoms and experienced (A) and anticipated (B) stigma

	MANSA	BPRS	DISC Mean Score A	DISC Mean Score B	DISC Count Score A	DISC Count Score B
Total sample (*n* = 171)						
Social integration	0.20	− 0.11	− 0.06	0.02	− 0.05	0.06
Consumption	0.06	0.01	− 0.04	− 0.05	− 0.03	0.02
Productivity	0.05	− 0.05	0.10	0.13	0.12	0.18
Access to services	0.09	0.03	− 0.02	− 0.02	− 0.01	− 0.03
Political engagement	− 0.04	− 0.05	0.08	0.03	0.07	0.00
Total SInQUE	0.16	− 0.12	0.00	0.00	0.01	0.07
Psychosis sample (*n* = 106)						
Social integration	0.29	− 0.18	− 0.10	0.07	− 0.11	0.08
Consumption	0.11	0.00	− 0.08	0.00	− 0.05	0.07
Productivity	0.12	− 0.06	0.02	0.14	0.04	0.18
Access to services	0.14	0.03	0.00	0.08	0.00	0.11
Political engagement	− 0.05	0.01	0.14	0.13	0.15	0.08
Total SInQUE	0.25	− 0.19	− 0.04	0.08	− 0.03	0.13
CMD sample (*n* = 33)						
Social integration	0.30	− 0.17	0.03	− 0.02	0.02	0.13
Consumption	**0.38**	− **0.54**	0.11	− 0.31	− 0.16	− 0.18
Productivity	0.06	− 0.06	0.30	0.15	0.30	0.25
Access to services	0.15	− **0.31**	− 0.11	− 0.41	− 0.14	− 0.33
Political engagement	0.07	− **0.33**	0.04	− 0.09	0.01	− 0.08
Total SInQUE	0.29	− **0.45**	0.05	− 0.15	− 0.01	− 0.05
PD sample (*n* = 32)						
Social integration	− **0.38**	0.51	0.11	0.01	0.13	0.05
Consumption	− **0.35**	**0.51**	0.17	− 0.04	0.17	0.01
Productivity	− 0.27	0.20	**0.38**	0.24	0.32	0.16
Access to services	− 0.17	0.27	− 0.04	0.09	0.09	0.23
Political engagement	− 0.03	− 0.14	− 0.11	− 0.28	− 0.13	− 0.25
Total SInQUE	− **0.43**	**0.57**	0.14	0.03	0.15	0.12

*r* > 0.3 considered significant (in bold)

### Measures of reliability

The T2 SInQUE showed good test–retest reliability with ICCs for all domains and the total score above 0.7. However, the ICCs for the T1 component fell below 0.7 for the service access and political engagement domains. Inter-rater reliability was excellent for all domains at T1 and T2 (Table 6).

**Table 6:** Test–retest and inter-rater reliability, values are ICCs (95% CI)

	T1	T2
Test–retest reliability (*n* = 30)		
Social integration	0.85 (0.71, 0.93)	0.90 (0.81, 0.95)
Consumption	0.89 (0.78, 0.94)	0.84 (0.69, 0.92)
Productivity	0.89 (0.79, 0.95)	1.0
Access to services	0.38 (0.02, 0.65)	0.78 (0.58, 0.89)
Political engagement	0.67 (0.41, 0.83)	0.95 (0.90, 0.98)
Total SInQUE	0.85 (0.71, 0.93)	0.92 (0.85, 0.96)
Inter-rater reliability (*n* = 11)		
Social integration	0.98 (0.92, 0.99)	0.98 (0.92, 0.99)
Consumption	1.0	0.98 (0.92, 0.99)
Productivity	0.99 (0.96, 1.00)	1.0
Access to services	1.0	1.0
Political engagement	0.84 (0.54, 0.96)	1.0
Total SInQUE	0.99 (0.97, 1.00)	0.98 (0.93, 0.99)

ICC > 0.7 considered acceptable

#### Acceptability to service users

Over 87% (192/220) eligible potential service user participants who were contacted by the researchers agreed to participate in the study. Of the 192 service users and 28 siblings recruited, none of them declined to complete the SInQUE or expressed concerns about its content or duration and there were no missing data in the scoring of the SInQUE. There were no missing data. This suggests that the tool is feasible for use with and acceptable to mental health service users.

#### Perceived utility to staff

Mental health and social care professionals who participated in the focus groups were generally positive about the SInQUE as a potentially useful tool to increase their understanding of their clients’ social inclusion. Staff felt that the SInQUE could be used to assess and monitor social inclusion, to facilitate discussions with their clients and guide care planning that could target specific aspects of social exclusion where greater support was needed. They also suggested that the measure could be useful for staff working in non-statutory services.

Staff suggested that there should be clear guidance on the timing of administration of the SInQUE for staff in different settings. The general view was that it would be important to complete it with service users who were in a more stable phase of illness, rather than when they were acutely unwell. Staff felt that the tool should not be completed too often in individual meetings with service users to ensure that there was enough time to allow progress to be made and to reduce any unnecessary burden on clinicians’ time.

## Discussion

The SInQUE (Social Inclusion Questionnaire, User Experience) assesses social inclusion in mental health service users, suffering from a wide range of psychiatric diagnoses, in a secondary care context. The measure appears to be acceptable to service users, based on the high rate of response and completion of the measure by service users, and is considered to have potential utility by mental health service staff as a clinical tool. We found that the component of the tool that assesses service users’ current social inclusion (T2) has good convergent and discriminant validity, test–retest reliability, and excellent inter-rater reliability. Contrary to expectations, however, there was no association between current levels of social inclusion and symptom severity or unmet needs. We would, therefore, only recommend the T2 part of the measure, which assesses current social inclusion, as a research measure or clinical tool to assess and monitor social inclusion and as an aid to care planning to assist in identifying specific areas of social exclusion where focussed interventions are needed.

This study confirmed our previous finding that social inclusion decreases after developing a mental illness. In this study, this held true when shared family and socio-cultural factors were accounted for [19]. Whilst our service user participants and their mentally well siblings had similar levels of social inclusion prior to the onset of the illness, service users became less socially included than their healthy siblings in three areas over time: social integration, productivity, and consumption. The slight increase in service users’ social integration domain score between the first (T1) and second (T2) time points, could be due to the input received from mental health and social care services with regard to accommodation and occupation or may, as with our sibling sample, simply reflect an increase in social integration, from adolescence to adulthood.

Of note, service users’ scores on the social integration domain in the year prior to their first contact with mental health services were lower than their siblings’, which might suggest that some had been experiencing difficulties affecting their social inclusion prior to their first admission to hospital/contact with a crisis team. This highlights the difficulty of identifying an ‘index event’ to identify the onset of illness, for the purpose of assessing social inclusion at T1. Given the number of years that most participants had been in contact with mental health services, we aimed to use a memorable event, such as first admission or first episode of treatment from a crisis team, to assist recall. However, not all participants had had these interventions. This qualification only relates to the T1 component of the tool. The high response and completion rates of the SInQUE appear to indicate that it is acceptable to service users.

### Strengths and limitations

Although all participants in the study had severe mental health problems, the most mentally unwell individuals may have been excluded from the study, due to concerns by their care co-ordinators about their ability to participate and/or their lack of capacity to give informed consent for participation. It may be that these individuals are at the greatest risk of social exclusion, given the trend for more severe symptoms being associated with low levels of social inclusion.

This study has not looked at the social inclusion of individuals with less severe mental health problems who are treated in primary care or other settings outside of statutory community mental health services. Further investigation of the acceptability and utility of the tool in these settings is warranted.

We do not know how the SInQUE scores in study participants compare with the general population, as no normative data exist. The non-affected siblings recruited to this study cannot necessarily be assumed to be representative of the general population because of the potential impact of having a sibling with severe mental illness on their own social inclusion.

Whilst the SInQUE domains have a theoretical basis. Their validity has not yet been confirmed through factor analysis and this remains a goal for future research with a larger sample.

We have explored the utility and acceptability of the measure to mental health staff through focus groups, but in this paper, have only assessed acceptability to service users through review of recruitment and completion rates. It would be important in any future study to be able to explore further the acceptability and perceived utility for service users as well as staff and the circumstances in which the SInQUE may inform and improve patient care. It would also be important to conduct an implementation study of the SInQUE with a range of community mental health services and to explore potential barriers and facilitators to using the SInQUE in clinical practice.

## Conclusion

The Social Inclusion Questionnaire User Experience (SInQUE) is a measure of social inclusion that can be used with mental health service users. The component of the tool that assesses current social inclusion has good psychometric properties and can be recommended as a research measure and for use by mental health staff to monitor their clients’ social inclusion and identify areas of social exclusion where specific care planning is required.
